# The Chemorepulsive Protein Semaphorin 3A and Perineuronal Net-Mediated Plasticity

**DOI:** 10.1155/2016/3679545

**Published:** 2016-01-14

**Authors:** F. de Winter, J. C. F. Kwok, J. W. Fawcett, T. T. Vo, D. Carulli, J. Verhaagen

**Affiliations:** ^1^Netherlands Institute for Neuroscience, Royal Academy of Arts and Sciences, Meibergdreef 47, 1105 BA Amsterdam, Netherlands; ^2^John van Geest Centre for Brain Repair, University of Cambridge, Cambridge CB2 OPY, UK; ^3^Department of Neuroscience, Neuroscience Institute of Turin (NIT) and Neuroscience Institute Cavalieri-Ottolenghi (NICO), University of Turin, Orbassano, 10043 Turin, Italy

## Abstract

During postnatal development, closure of critical periods coincides with the appearance of extracellular matrix structures, called perineuronal nets (PNN), around various neuronal populations throughout the brain. The absence or presence of PNN strongly correlates with neuronal plasticity. It is not clear how PNN regulate plasticity. The repulsive axon guidance proteins Semaphorin (Sema) 3A and Sema3B are also prominently expressed in the postnatal and adult brain. In the neocortex, Sema3A accumulates in the PNN that form around parvalbumin positive inhibitory interneurons during the closure of critical periods. Sema3A interacts with high-affinity with chondroitin sulfate E, a component of PNN. The localization of Sema3A in PNN and its inhibitory effects on developing neurites are intriguing features and may clarify how PNN mediate structural neural plasticity. In the cerebellum, enhanced neuronal plasticity as a result of an enriched environment correlates with reduced Sema3A expression in PNN. Here, we first review the distribution of Sema3A and Sema3B expression in the rat brain and the biochemical interaction of Sema3A with PNN. Subsequently, we review what is known so far about functional correlates of changes in Sema3A expression in PNN. Finally, we propose a model of how Semaphorins in the PNN may influence local connectivity.

## 1. Introduction

Since the early 1990s, chemorepulsion has been recognized as an effective way to guide growing neurites towards their targets in the developing nervous system [1]. One of the protein families that has been extensively studied in the context of repulsive axon guidance is the Semaphorin family (reviewed in [2, 3]). Although the secreted and transmembrane Semaphorins were initially identified as repulsive axon guidance cues [4], in the past decade they have also been linked to many other cellular processes, including cell migration, proliferation, and polarization. Because of their role in these key cellular functions they also have been implicated in various diseases, including cardiovascular and immunological diseases and cancer [5, 6]. In addition, the repulsive properties of especially the secreted Semaphorins are thought to hamper regenerative processes following nervous system trauma [7–9]. Recently, studies have implicated Semaphorins in synaptic and structural plasticity (reviewed in [10]) and in certain neurological diseases (reviewed in [11]).

Invertebrate, vertebrate, and viral Semaphorins all share a conserved Semaphorin domain. Semaphorins are divided into 8 classes, based on their structure and sequence similarities [12]. All transmembrane and secreted Semaphorins, except for Sema7A, use Plexins as signal transducing receptor. However, many Semaphorins make use of additional receptor components. For instance, all class 3 Semaphorins, except for Sema3E, require Neuropilin as ligand-binding receptor component to achieve repulsive guidance signaling through Plexins [13–18]. Other than Plexins, VEGFR2, ErbB2, and IgCAM can also be part of different Semaphorin receptor complexes (reviewed in [19]). The outcome of Sema-Plexin signaling is, however, highly context dependent. For example, class 5 Semaphorins demonstrate repulsive properties on neurites that express chondroitin sulfate proteoglycans (CSPGs) adjacent to Plexins while these same Semaphorins turn into an attractive cue if neurites coexpress heparan sulfate proteoglycans (HSPGs) with Plexins on their surface [20]. Binding of Semaphorin to its receptor complex starts an intrinsic signaling pathway that involves GTPases and the PI3K/Akt pathway and eventually leads to reorganization of the cytoskeleton [21–24]. In addition, Semaphorin stimulation leads to an activation of the Plexin interacting protein MICAL (molecule interacting with casL) and to the phosphorylation of intracellular proteins of the CRMP (collapsing responsive mediator protein) family, which in turn affects F-actin and microtubule disassembly and thereby growth cone motility [25–31].

Next to their role in long distance axon guidance during development of the nervous system, Semaphorins also play a role in local target selection (reviewed in [10]). Recent studies have shown that class 3 Semaphorins/Plexins complex regulates connectivity not only at a cellular but also at a subcellular level such as the formation of synapse and spine. In the developing spinal cord reflex circuitry, Sema3E is only expressed by motoneurons that innervate the cutaneous maximus muscle but not by motoneurons that innervate the triceps muscle [32]. PlexinD1 expressing sensory axons of the triceps muscle form direct synaptic contacts with their Sema3E negative motoneurons. The PlexinD1 positive sensory afferents of the cutaneous muscle are however repelled by Sema3E expressing cutaneous maximus motoneurons. As a result, sensory and motor neurons of the cutaneous muscle cannot form direct but only indirect synapses. Knockout of either PlexinD1 in the sensory afferents or Sema3E in the motoneurons does allow the formation of direct synaptic contacts between cutaneous sensory and cutaneous motoneurons. In addition, the Sema3E-PlexinD1 interaction specifically determines the number of glutamatergic connections formed between Sema3E expressing thalamostriatal projection neurons and the PlexinD1 positive direct pathway medium spiny neurons but not the PlexinD1 negative indirect pathway medium spiny neurons [33].

At a subcellular level, several Semaphorin family members have been shown to control multiple aspects of synapse formation during late development of the mammalian nervous system. Sema3A increases the clustering of pre- and postsynaptic proteins in cortical neurons* in vitro* without affecting the number of spines [34, 35] while Sema3F limits the number of dendritic spines distributed along the apical dendrites of pyramidal neurons in the cerebral cortex, thereby locally restricting the options for synapse development on the apical dendrite [36, 37]. In the hippocampus, Sema5A limits the number of spines formed on the dendrites of developmentally and adult born granular neurons in a cell autonomous way. Mutant mice for Sema5A or its receptor PlexinA2 display an increase in glutamatergic synapses [38]. In addition, the transmembrane class 4 Semaphorins, Sema4B and Sema4D, have been shown to regulate GABAergic and glutamatergic synapse numbers in cultured dissociated hippocampal neurons [39–41]. Also in the developing cerebellum, Semaphorins control the number of synapses. Sema3A and Sema7A control the different stages of synapse elimination. In Purkinje cells knockdown Sema3A leads to an acceleration in synapse elimination while a knockdown of Sema7A results in an impairment of synapse elimination [42]. In addition, the diffusible fragments of processed Sema5B have the capacity to induce synapse elimination in cultured hippocampal neurons [43]. Besides their guidance role in neuronal circuit formation during development, Semaphorins can modulate the functional properties of established synapses in the adult brain. Sema3F application to adult hippocampal slices induces an increase in frequency and amplitude of excitatory postsynaptic currents in dentate granule neurons and CA1 pyramidal neurons [44]. However, application of Sema3A to the hippocampus results in a reduction in synaptic efficiency [45].

**Figure 1:**
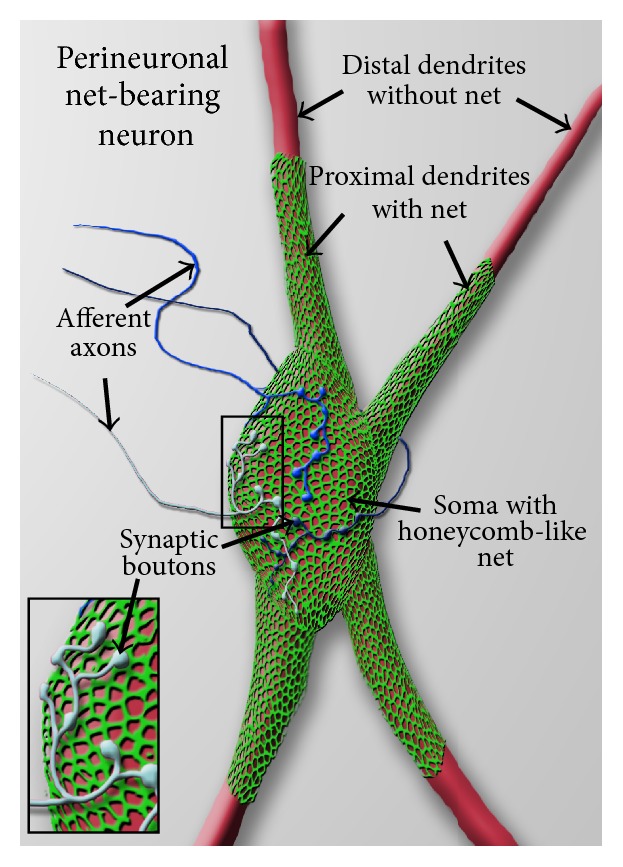
Schematic view of a perineuronal net-bearing neuron. A neuronal cell body (soma) with its proximal dendrites (red) covered by a typical reticular, honeycomb like net (green). More distally, dendrites are devoid of nets. The holes in the perineuronal nets are occupied by synaptic boutons of afferent axons (insert, blue) that synapse on the net-bearing neuron.

The continuous expression of some Semaphorins even into adulthood indicates that Semaphorins may be involved in sculpturing the neuronal circuits beyond the initial development of the nervous system [46]. A structure that is thought to play an important role in adult central nervous system neuronal plasticity is the perineuronal net (PNN; Figure 1) [47]. PNNs are highly condensed extracellular matrix aggregates that form around the cell bodies and proximal dendrites of many populations of neurons during the closure of a critical period [48, 49]. Recent work shows that the chemorepulsive proteins, Semaphorin 3A and Semaphorin 3B, are highly concentrated in the PNNs of distinct neuronal populations in the maturing brain and are therefore in an excellent position to control PNN-mediated neuronal plasticity during and/or beyond the critical period [50, 51]. In the following sections, we summarize the localization of Semaphorin 3A protein in the adult rodent brain [51], review the biochemical data on the interaction of this protein with the extracellular matrix [52], and discuss the possible role of Semaphorin 3A and Semaphorin 3B in PNN-controlled structural plasticity.

## 2. Semaphorin Expression in the Mature Brain

**Figure 2:**
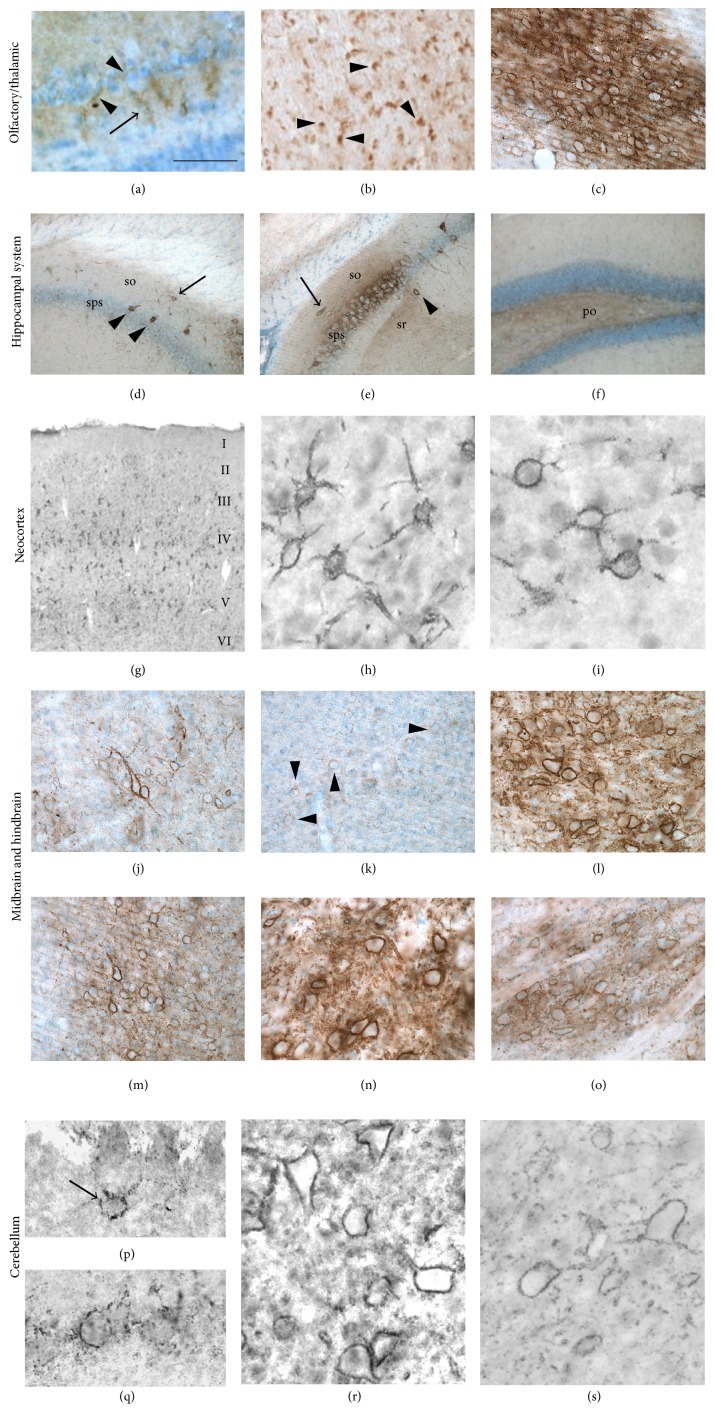
Sema3A expression in the rat brain from rostral to caudal. In the olfactory bulb (a), faint Sema3A-immunoreactive labeling was detected between mitral cells (arrowheads) as well as around the initial segments of the mitral cell dendrites in the external plexiform layer (arrow). A coronal section of the lateral olfactory tract (b) showed strong Sema3A-immunostaining in myelinated axons (arrowheads). In the reticular thalamic nucleus (c), intense diffuse labeling is present throughout the nucleus. The neurons in the reticular thalamic nucleus are surrounded by strongly Sema3A-labelled PNNs. Sema3A immunoreactivity in the hippocampus was observed in various structures. Interneurons in the stratum oriens of CA1 (so, arrow) (d) and in the stratum oriens (so, arrow) and stratum radiatum (sr, arrowhead) of CA2 (e) displayed Sema3A positive PNNs. Only the pyramidal neurons in CA2 and not in CA1 and CA3 displayed striking intense Sema3A-labelled PNNs with strong diffuse labeling between the cell bodies of pyramidal neurons (sps) (f). The polymorph layer (po) of the dentate gyrus displayed a weak diffuse staining for Sema3A. In the neocortex, neurons with a Sema3A-positive PNN are scattered throughout layers II to VI (g). This typical Sema3A labeling in the cortical layers was observed in various anatomical subdivisions of the neocortex, including the somatosensory cortex (h) and the visual cortex (i). Well defined Sema3A-labelled PNNs are present around neurons in the deep gray of the superior colliculus (j); this is in contrast to the weakly labelled PNNs surrounding the neurons of the intermediate gray of the superior colliculus (arrowheads) (k). The red nucleus shows a large number of strongly Sema3A-labelled PNNs around the magnocellular neurons. In addition, an intense diffuse Sema3A staining was observed between neurons (l). Also the external nucleus of the inferior colliculus displayed strong and well-defined Sema3A immunoreactive PNNs and strong diffuse labeling between the neurons throughout the nucleus (m). The vestibular nucleus of the hindbrain contains many large neurons with robust Sema3A-positive PNNs and strong diffuse immunoreactivity between the cells in the nucleus (n). Moderately Sema3A-labelled PNNs were observed around neurons in the inferior olive while throughout the nucleus a diffuse Sema3A labeling was present (o). In the cerebellum, some Golgi neurons bear Sema3A-positive PNNs ((p), arrow). Many Purkinje cells displayed a semiorganized Sema3A-pattern which is typical for the PNNs found around Purkinje cells (q). Deep cerebellar nuclei display well-defined large Sema3A and Sema3B immunoreactive neurons (resp., (r) and (s)). Images (d)–(s) have been reproduced with permission from Vo et al. [51]. Scale bar for (a) and (b) is 25 *µ*m; for (c) and (j)–(o) is 100 *µ*m; for (d)–(f) is 200 *µ*m; for (g) is 300 *µ*m; for (h) and (i) is 33 *µ*m; and for (p)–(s) is 35 *µ*m.

The persistent expression of Sema3A in the mature rat and human brain was initially shown at the mRNA level [46]. Neurons in the olfactory system, the cerebral cortex, and entorhinal-hippocampal system exhibited high levels of Sema3A mRNA expression. Extensive characterization of recently developed commercial antibodies against Sema3A and Sema3B has led to the identification of several antibodies that allowed reliable protein localization studies of Sema3A and 3B in the rodent nervous system [51]. Immunohistochemistry for Sema3A confirmed the ongoing expression of Sema3A at the protein level in the adult rat and mouse brain [51]. Several specific brain structures along the rostral-caudal axis, including the olfactory system, hippocampus, and cerebral cortex, show distinct patterns of Sema3A-immunoreactivity. In general, the immunoreactivity of Sema3A varied from a diffuse signal in between subsets of neurons, to a highly concentrated signal that appears as a reticular structure around the cell bodies and proximal dendrites of individual neurons (Figure 2). Sema3B-immunoreactivity shows a signal with similar features but its expression throughout the brain is more restricted [51].

In the* olfactory system*, Sema3A is diffusely expressed around some mitral cells and their dendrites in the outer plexiform layer (Figures 2(a) and 2(b)). The inner plexiform and granule cell layers have only weak diffuse Sema3A expression while the deeper portions of the granular cell layer are devoid of any immunoreactivity. Clear Sema3A positive PNN structures are present in the ventral anterior telencephalon, especially in layer III of the taenia tecta and the piriform cortex. Also the axonal projections of mitral cells through the lateral olfactory tract are Sema3A positive. In contrast to Sema3A, Sema3B expression is not detected in the main olfactory bulb [51].

In the* thalamic region*, PNN-like structures in the medial septal nucleus and the reticular nucleus show strong Sema3A immunoreactivity (Figure 2(c)). In addition, an intense but diffuse staining is present in between the cell bodies throughout the entire reticular nucleus. A similar but less intense staining is seen between the neurons in the zona incerta. The zona incerta also contains small groups of neurons with Sema3A positive PNNs. The stria medullaris of the thalamus and the optic chiasm have Sema3A-positive axons. Sema3B-immunoreactivity in the thalamic area is restricted to the reticular nucleus [51].

In the* hippocampal system*, numerous neurons have a PNN-like Sema3A immunoreactivity while in the indusium griseum, fasciola cinerea, and the stratum oriens of CA1, the pyramidal layer of CA3 and in the dentate gyrus granular cell layer only occasional Sema3A positive PNNs are observed (Figures 2(d)–2(f)). The pyramidal cells in CA2 have remarkable strong Sema3A-labelled PNNs and also intense diffuse labeling between the cell bodies. The neurons of the stratum oriens and stratum radiatum of the CA2 and CA3 and the polymorph layer of the dentate gyrus have a more diffuse Sema3A staining. Sema3B expression in the hippocampus is much more restricted than Sema3A. Sema3B positive PNNs are only present in the subiculum and weak diffuse labeling is seen in the CA1, CA2, and CA3 pyramidal cell layer [51].

Both Sema3A and Sema3B are prominently expressed in the PNN of numerous neurons in all subareas of the* neocortex* (Figures 2(g)–2(i)). Neurons with Sema positive PNNs are distributed scatteredly in the infralimbic, cingulated, parietal association, motor, somatosensory, agranular, gustatory, retrosplenial, auditory, and visual cortex. Most prominent expression exists in the cortical layers IV and V, except for the parietal association cortex and the anteromedial visual cortex where most Sema labeling is present in the deeper portion of layer V.

Several nuclei in the* midbrain* express Sema3A in PNN-like structures (Figures 2(j)–2(l)). There are strongly Sema3A-labelled PNNs on numerous neurons in the nucleus of Darkschewitsch and in the deep layers of the superior colliculus, whereas the intermediate layers of the superior colliculus contain moderately Sema3A-labelled PNNs and the superficial layers only contain weakly labelled PNNs. The stratum zonale has only weakly stained PNNs. The magnocellular neurons of the red nucleus have clear dense Sema3A staining in their PNNs while the staining between the neurons is more diffuse. Also in the inferior colliculus the PNNs are strongly Sema3A-labelled with a diffuse signal between neurons. In the tegmental reticular nucleus and in the nuclei of the lateral lemniscus, Sema3A is located around and between subpopulations of neurons. Sema3B-labeling largely overlaps with that of Sema3A. Sema3B stained PNNs are apparent in the deep gray layer of the superior colliculus and many neurons in the red nucleus [51].

In the* hindbrain,* Sema3A and Sema3B positive PNNs are heterogeneously present throughout the lateral, spinal, and medial vestibular nuclei (Figures 2(m)–2(o)). In addition, these nuclei have a strong diffuse immunoreactivity. Also large neurons in the pontine nucleus and in the superior and inferior olive have Sema3A and Sema3B labelled PNNs [51].

Many Purkinje cells in the* cerebellum* are surrounded by thin and granular Sema3A-positive PNNs (Figures 2(p)–2(r)). This is in contrast to the deep cerebellar nuclei, including the interpositus, fastigial, and dentate nuclei, where they all have a very intense Sema3A-signal around their neurons and a moderately diffuse staining throughout the nuclei. Sema3B is also expressed around and between the neurons of the deep cerebellar nuclei (Figure 2(s)).

## 3. Semaphorin 3A Is a Component of Perineuronal Nets

Double labeling for Sema3A and the classical PNN marker* Wisteria floribunda* agglutinin (WFA) indicates that Sema3A is located in the PNNs surrounding neurons [51]. In the PNN, Sema3A colocalizes with known individual components of PNN, including various CSPGs such as versican, aggrecan, phosphacan, and the glycoprotein tenascin-R. The presence of Sema3A around neurons depends directly on intact PNN. Enzymatic digestion of chondroitin sulfates, a key component in the PNNs,* in vivo* strips the neurons not only from their WFA positive nets but also from Sema3A protein. Recent work has shown that hyaluronan and proteoglycan link protein 1 (hapln1; also known with an alternative name as cartilage link protein 1) is necessary for normal PNN formation. Brain specific hapln1-mutant mice form only vestigial PNNs around neurons that normally have well-developed PNN [55]. In these mice with attenuated PNN, virtually no Sema3A-positive PNN are found [50, 51]. The colocalization of Sema3A with WFA and multiple CSPGs and the decline of Sema3A-positive PNN after enzymatic or genetic perturbation of the PNNs demonstrate that this repulsive axon guidance protein is a genuine component of PNN.

**Figure 3:**
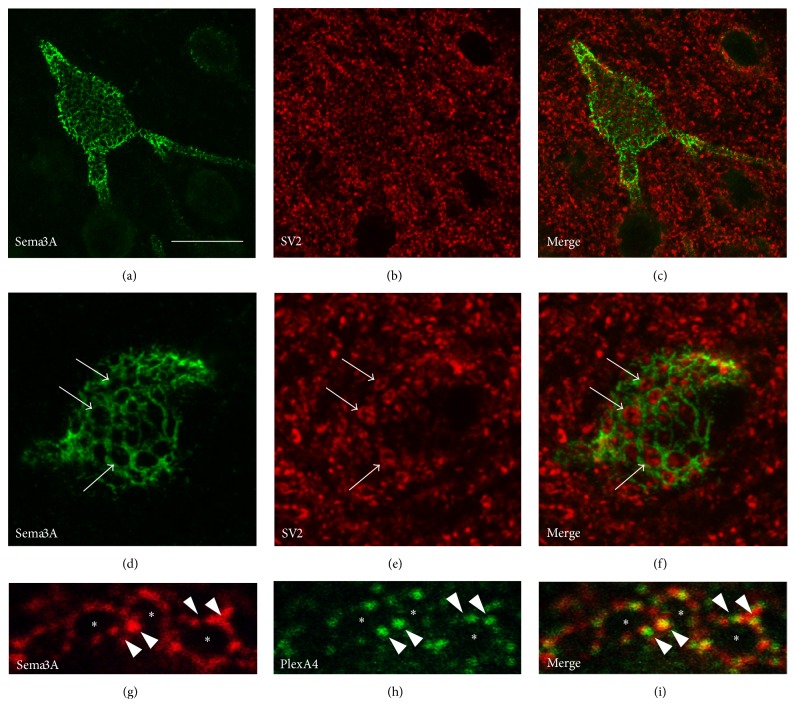
Sema3A expression in PNN. Confocal microscopy shows Sema3A immunoreactivity (green) which surrounds the somatodendritic compartment of an interneuron in the rat neocortex. Sema3A staining displays a honeycomb-like pattern which is typical for the structure of PNNs (a)–(c). Colabeling with the presynaptic marker SV2 (red) reveals that synaptic boutons on the neuron surface fill the holes in the Sema3A-positive honeycomb-like structure of the PNN ((d)–(f), arrows). The Sema3A receptor component PlexinA4 (green) is closely associated with the Sema3A (red) positive net. Like Sema3A, PlexinA4 (arrowheads) is not present in the holes (asterisks) of the honeycomb-like structure of the PNN (g)–(i). Scale bar for (a)–(c) is 20 *µ*m; for (d)–(f) is 8 *µ*m; for (g)–(i) is 3.5 *µ*m.

In the adult brain, pericellular Sema3A staining was only observed around cells that do also have a WFA positive PNN. However, not all WFA-positive neurons have Sema3A-immunoreactivity in their PNN, indicating that the presence of Sema3A in PNN is restricted to a subset of PNN bearing neurons. In the neocortex, most PNN are found around parvalbumin (PV) and calbindin positive inhibitory interneurons [56]. Double staining for these interneuron markers and Sema3A revealed that, in the rat and mouse visual cortex, Sema3A is only present in the PNNs around PV-positive interneurons. High magnification images showed that Sema3A positive PNNs form a honeycomb-like structure around the soma and proximal dendrites of the PV-cells (Figure 3). The holes in these honeycomb-like PNNs are occupied by presynaptic terminals that synapse on the PV-interneurons. This means that the localization of Sema3A in the PNN brings it not only in close contact with the PV-interneuron itself but also with the presynaptic neurons that form synapses on the somatodendritic compartment of the PV-interneuron. Sema3A receptors have been shown to be expressed by PV-interneurons of the visual cortex. Sema3A in the PNN is closely associated with PlexinA1 and A4 positive microdomains on the PV-interneurons cell membrane. This close contact is suggestive of direct signaling between Sema3A in the PNN and the PV-interneuron. It is currently not clear if the synaptic terminals on the PV cell body also express Sema3A receptor components.

The cellular source of Sema3A protein in PNN is not known. Sema3A protein might be locally produced or it could be produced elsewhere in the brain and subsequently transported to the PNNs. Comparing results from* in situ* hybridization studies with immunohistochemistry studies for Sema3A reveals that there is no direct correlation between Sema3A mRNA expression and the presence of a Sema3A-positive PNN around a particular neuron. Both Sema3A mRNA expressing and nonexpressing neurons can have a Sema3A-positive PNN. Moreover, neurons that do express Sema3A mRNA but do not have a Sema3A bearing net exist. The absence of a clear overlap between mRNA expression and the presence of Sema3A protein in the PNN suggest that Sema3A is produced elsewhere, being transported and presented to the PNN. Previous studies have shown that in embryonic rat cortical neurons, endogenously or exogenously expressed Sema3A is actively transported in vesicles through both the axon and dendrites of the cell [57, 58]. In axons, Sema3A is almost exclusively transported in an anterograde direction. This transport is activity dependent, whereby depolarizing the cell results in an arrest and action potential blocking in an acceleration of axonal transport of Sema3A. Moreover, depolarization also induced the delivery of Sema3A at the cell surface as a stable deposit [58]. Taken together, this suggests a model in which presynaptic neurons produce Sema3A, transport the protein to their terminals, and subsequently deposit it in the PNN of PV-interneurons in an activity dependent manner.

Alternatively, Sema3A could be produced and secreted by nonneuronal cells in the brain. Previous studies showed that astrocytes do not express Sema3A and these cells can therefore be excluded as a source of Sema3A [46]. Recently, it has been shown that Orthodenticle homeobox 2 (OTX2), another protein that binds to the PNN, is produced by cells in the choroid plexus and released in the cerebral fluid [54, 59]. From the cerebral spinal fluid, OTX2 is able to travel and bind to the PNN in the neocortex. Conditional removal of OTX2 expression in choroid plexus cells results in PNNs that are devoid of OTX2 in the neocortex [59]. This shows that choroid plexus cells are indeed an unexpected source of OTX2 in PNN. Like OTX2, Sema3A is highly expressed by choroid plexus cells in the ventricles (unpublished observations). In addition, meningeal cells surrounding the brain express high levels of Sema3A [7]. Similar to OTX2 [59], Sema3A may be released by cells of the choroid plexus or the meninges in the cerebral spinal fluid and travel through the brain to bind to the PNNs.

## 4. Biochemical Properties of Sema3A-PNN Interactions

**Figure 4:**
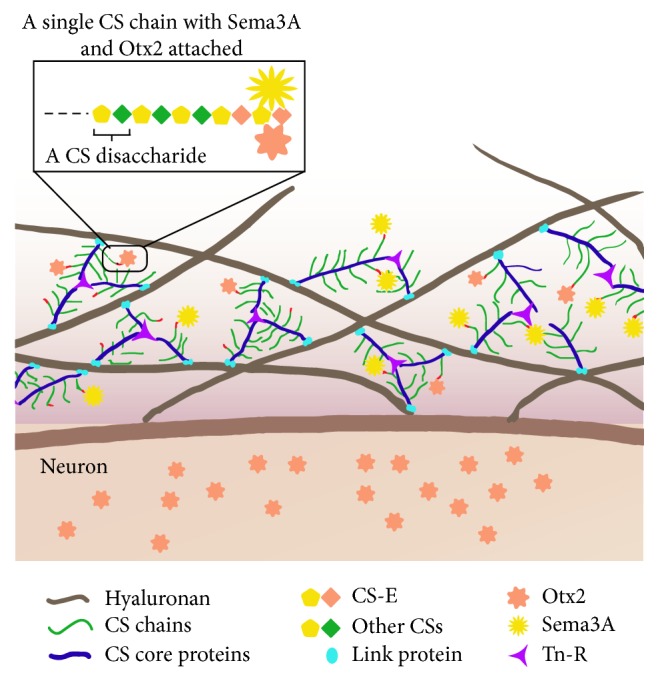
Schematic representation of Sema3A protein localization in PNNs. Hyaluronan molecules attached to the neuron cell membrane contain link proteins that carry the CS chain bearing CS core proteins. Tn-R act as a cross-linking protein between several CS core proteins present on different hyaluronan molecules. Both Sema3A and OTX2 preferentially bind to the CS-E chains attached to the CS core proteins. After binding to the PNN via CS-E, OTX2 can translocate into the neuron [53, 54], whereas Sema3A appears to reside in the PNN using CS-E as “an anchor.”

The interaction of Sema3A with PNNs is mediated through a specific glycan structure called chondroitin sulfate (CS) (Figure 4). In order to understand the molecular interaction between Sema3A and PNNs, a more detailed understanding of PNNs and their molecular composition is crucial. PNNs are stable macromolecular aggregates of brain extracellular matrix molecules tethered on the surface of subpopulations of neurons [60–62]. They are composed of CSPGs, tenascin-R, and members from the Hapln family [63, 64]. Both CS and hyaluronan belong to the family of glycosaminoglycans (GAGs), of which the linear GAG chains are covalently attached to a protein core forming proteoglycan (PG) [65]. The number of disaccharides in a single CS-GAG chain and the number of CS-GAG chains attaching to a core protein are highly variable. The complexity of CS GAGs is further heightened by the addition of sulfate groups at various locations in the disaccharide subunits, resulting in wide heterogeneity of CSs, of which CS-A, -C, -D, and -E are the most common isoforms [66].

The first indication of Sema3A interacting with CSs comes from a study by De Wit et al. [67]. With the use of Neuro2a cells, it has been shown that the addition of excess GAGs including CS-A and CS-B, heparan sulfate and heparin to the culture medium is able to displace the surface bound Sema3A. Enzymatic removal of CSs using chondroitinase ABC (ChABC), but not of heparan sulfate by heparinase III, releases Sema3A-GFP to the culture medium. This suggests Sema3A is indeed bound to CSs on the cell surface. Further evidence for a direct interaction of Sema3A and CS is shown by immunoprecipitation of Sema3A in embryonic brain extract, in which CS is visualized using a pan-CS antibody [68].

The biological properties of CSPGs are heavily dependent on the attached CS GAG chains. Digestion of the CS GAGs using ChABC in the inhibitory glial scar formed after spinal cord injury has rendered the environment more permissive to axonal growth and regeneration [69–71]. While ChABC removes all CS isoforms, recent study suggests that the different CS isoforms impose distinct effects on neuronal growth [72–74]. Knockout mice with reduced level of 6-sulfated CS (CS-C) demonstrate worse regeneration in nigrostriatal axons when compared with wild type mice [73]. This inferior regeneration ability is, however, not observed in a peripheral nerve injury system due to a compensatory upregulation of 6-sulfates in the PNS. Increased expression of 6-sulphated CS in animals that overexpress the 6-sulfotransferase enzyme is associated with increased plasticity, which persists into adulthood [74]. On the contrary, chondroitin 4-sulfates (CS-A) are strongly upregulated after spinal cord injury and have been shown to inhibit axonal growth in cultured cerebellar granular neurons, suggesting a diversity of functions from various sulfated CSs [72, 75]. An analysis of CS sulfation composition in an adult brain has demonstrated differences between the GAGs recovered from the PNNs or from the loose brain extracellular matrix (ECM) in an adult rat brain [76]. While 4-sulfation (CS-A) is the predominant CS isoform in an adult brain, the percentage has however dropped from ~90% in the ECM GAGs to ~80% in the PNN GAGs. On the contrary, both the 6-sulfation (CS-C) and the 4,6-disulfation (CS-E) are increased in the PNN GAGs [76]. These experiments prompt a question: could the interaction of Sema3A with PNNs be sulfation specific? The binding of several growth factors and morphogens has been shown to be sulfation pattern dependent [77]. With the use of a modified enzyme-linked immunosorbent assay, carbohydrate microarrays, and proteomic analysis, it has now been confirmed that the interaction between Sema3A and PNNs is sulfation-dependent [52, 78]. Among the four most common CSs and heparan sulfate used in the assay, Sema3A interacts preferentially to CS-E in a concentration dependent manner. This binding is partially blocked by an anti-CS-E antibody [52]. CS-E is a disulfated CS structure, similar to CS-D, bearing two highly negatively charged sulfate groups. The lack of binding of Sema3A to CS-D but only to CS-E suggests that this interaction is specific and is not due to a nonspecific charge interaction.

The binding of homeoprotein OTX2 to the PNNs in PV positive neurons is also mediated through CS-E [54, 79]. Enzymatic digestion of CS or blocking the binding of OTX2 to the PNNs prevents the internalisation of OTX2, changes the expression of PV, and reopens ocular dominance plasticity in an adult visual cortex [54, 59]. A basic motif composed of a 15-amino acid motif enriched in arginine-lysine (RK) doublets at the N-terminal of OTX2 is responsible for OTX2 binding to PNNs [54]. Although a specific CS-binding domain has not yet been identified in Sema3A, Sema3A also contains a long basic domain at its C-terminus. Whether this basic domain is responsible for binding to the PNNs merits further investigation.

## 5. Functional Implications of Sema3A in PNNs

The effect of Sema3A binding to the PNNs in mature CNS neurons has yet to be elucidated. We could however get a glimpse of the potential function from the work in dorsal root ganglion (DRG) neurons or embryonic cortical neurons* in vitro*. Growth cones of embryonic DRG neurons were exposed to Sema3A collapse and this collapse is potentiated by heparin [67]. Adult DRG neurons cultured on isolated PNN-GAGs project shorter neurites than those being cultured on general brain GAGs, suggesting that PNN-GAGs are more inhibitory than general GAGs. This inhibition is further enhanced by the presence of Sema3A. Blocking Sema3A binding to PNN-GAGs reverses this inhibition [52]. Similar effects are also observed in E13 cortical culture. Cortical neurons avoid the area where CSPGs are present and this repellent property is strengthened in the presence of Sema3A [68]. These results suggest that CS may be involved in regulating the degree of inhibition of Sema3A on neuronal growth.

**Figure 5:**
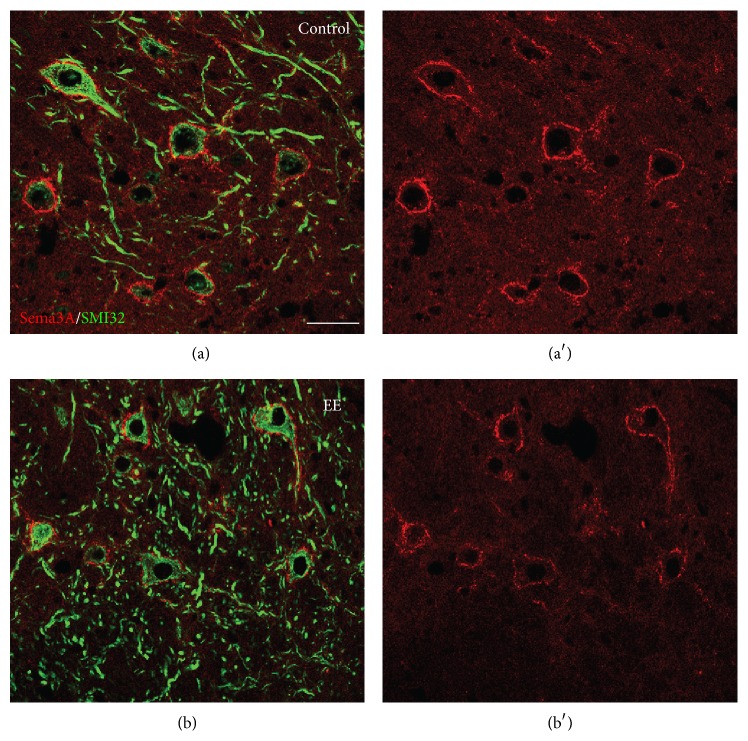
Exposure to enriched environment induces a reduction of Sema3A in the cerebellar nuclei of the adult mouse. (a)–(b′) show Sema3A immunostaining (red) around projection neurons of the cerebellar nuclei (stained by SMI32, green) of control mice (a, a′) and mice exposed to enriched environment (b, b′). A clear reduction of Sema3A levels is observed in enriched animals. EE: enriched environment. Scale bar: 50 *µ*m.

Based on these studies, it is hypothesized that Sema3A binding to PNN-GAGs may act as an inhibitory cue for presynaptic remodeling and formation of new connections on PNN-bearing neurons. To test this hypothesis, recent studies examined Sema3A expression in conditions associated with plasticity. For example, when adult mice are reared in an enriched environment (a condition known to strongly promote neuronal plasticity (see for reviews [80, 81])), a strong reduction of WFA-positive PNNs occurs in these mice and the Sema3A content of the PNN in the cerebellar nuclei decreases (Figure 5). In parallel, enhanced structural plasticity of Purkinje cell terminals and precerebellar afferents is observed [50, 82]. Sema3A levels in PNNs are also altered in the injured CNS during compensatory sprouting. Partial deprivation of cerebellar nuclei neurons of their main inputs, the Purkinje cells, results in a strong decrease of both CSPG and Sema3A labeling surrounding denervated neurons, in association with structural reorganization of the local connectivity [50]. These studies suggest that Sema3A in PNNs can be actively modulated to facilitate or restrict plasticity according to specific functional requirements. Interestingly, reduced Sema3A expression in PNNs is also observed in the cerebellar nuclei of transgenic mice selectively overexpressing the growth-associated protein GAP-43 in Purkinje cells [50], suggesting that enhanced intrinsic growth properties of Purkinje cells are accompanied by parallel modulation of extrinsic regulatory cues [83, 84]. Mice lacking Hapln1, which have attenuated PNNs with strongly diminished Sema3A levels, display persistent plasticity into adulthood [51, 55]. Collectively, these data suggest that Sema3A is an important functional attribute of PNNs, contributing to their growth-inhibitory properties.

In accordance with the idea that Sema3A may play a role in the regulation of the functioning of neuronal circuits in the adult brain, in an experimental model of temporal lobe epilepsy, which is characterized by robust sprouting of hippocampal mossy fibres in the molecular layer of the dentate gyrus, a transient downregulation of Sema3A mRNA in stellate neurons of the entorhinal cortex occurs. Entorhinal stellate neurons project their axons to the molecular layer of dentate gyrus and thus may secrete Sema3A into this region (which indeed is devoid of Sema3A mRNA) [85]. These data suggest that Sema3A derived from the entorhinal cortex may restrict structural changes in the molecular layer of the dentate gyrus throughout life. Also in the cerebellum, where Sema3A may be released by axon terminals of Purkinje cells, which express Sema3A mRNA, to regulate Purkinje cell specific connection patterns in the cerebellar nuclei. Indeed, Sema3A is tightly associated with Purkinje axons and their terminals and its amount in PNNs around cerebellar nuclei neurons is relation to Purkinje cell innervation of target neurons [50]. Similarly, Sema3A produced by primary sensory neurons regulates the number of sensory innervations of trabecular bone in an autocrine manner [86]. Also during development, Sema3A exerts a cell-autonomous effect on the outgrowth of axons, by regulating the sensitivity of motoneuron growth cones to exogenous Sema3A [87]. Moreover, in cultures of differentiated hippocampal neurons, Sema3A elicits a strong reduction of the size of pre- and postsynaptic structures [45]. Finally, inhibition from Sema3A on axon outgrowth of calcitonin gene related peptide- (CGRP-) positive sensory fibers is shown following injection of adenovirus encoding Sema3A in the spinal cord after complete spinal transection in a model of autonomic dysreflexia [88].

To exert an effect on PNN-bearing neurons or synaptic boutons, Sema3A must interact with the NP-1/Plexin receptor complex. In the cerebellar nuclei, where strong perineuronal Sema3A is present, both nuclear neurons and Purkinje cell axons express the Sema3A receptor component PlexinA4 [89]. NP-1 is strongly expressed in the molecular layer of the dentate gyrus, where entorhinal stellate neurons project their axons [85]. In the cerebral cortex, where Sema3A containing PNNs are mainly associated with GABAergic interneurons, PlexinA1 and PlexinA4 are concentrated in globular structures on the plasma membrane of those neurons. These Plexin-positive microdomains are closely associated with Sema3A that is concentrated in the PNN [51], suggestive of Sema3A-Plexin-mediated signalling between the PNN and the soma of the inhibitory neuron. Moreover, interneurons display abundant expression of flotillin-1 [51], a lipid raft protein that is essential for Sema3A induced growth cone turning and endocytosis [90].

The interaction of Sema3A with CSPGs could concentrate these cues at specific sites and/or potentiate or modify their activity. The possible additive effects of Sema3A with PNN-GAGs could be mediated by the independent signalling pathways these two families of molecules are eliciting. Whereas Sema3A signals through the NP-1/Plexin receptor complex, the inhibition from CSs in the PNN-GAGs may be triggered via CSPG receptors, including protein-tyrosine phosphatase-sigma, leukocyte common-related phosphatase, or Nogo receptor-1 or receptor-3 [91–93]. It is therefore possible that the simultaneous presentation of both Sema3A and CSs on the neuronal surface confers stronger inhibitory properties to the PNNs.

## 6. Conclusions and Perspective

PNNs around inhibitory interneurons play a key role in the regulation of the critical period. In many developing neural systems, the closure of the critical period shows a strong correlation with the appearance of PNNs around inhibitory neurons [94–97]. Preventing sensory input, like dark rearing, prolongs the period of ocular dominance plasticity and delays PNN formation in the visual cortex [94, 95]. Degradation (by chondroitinase ABC) or the formation of strongly attenuated PNN (as in Hapln1 mutant mice) restores or retains plasticity, respectively [55, 94, 96]. How the PNN regulates plasticity is still poorly understood, as it turns out to be very difficult to unravel the mechanistic cellular and molecular basis of PNN function. The discovery that chemorepulsive proteins like Sema3A and Sema3B are molecular components of PNN offers new avenues for research into how the PNN may influence neuroplasticity.

**Figure 6:**
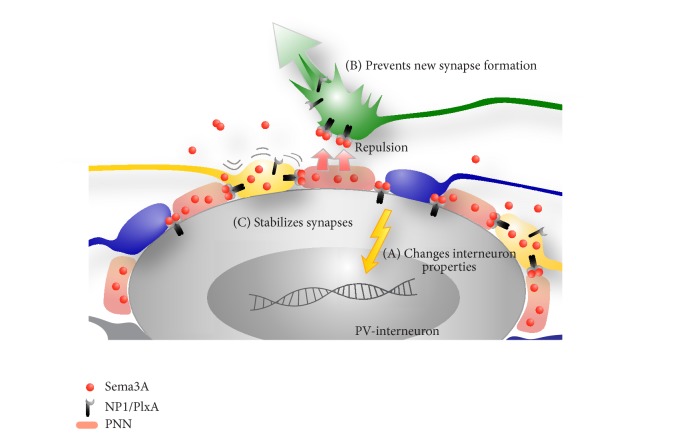
Schematic view of possible roles for Sema3A in PNN related plasticity. Sema3A protein molecules (red spheres) derived from the more distant cells in the environment, that is, meningeal cell or cells of the choroid plexus, or secreted along axons or by presynaptic terminals (yellow) integrate in the PNN surrounding parvalbumin (PV) positive interneurons. PV-interneurons express Sema3A receptor components (NP1/PlxA) which may trigger an internal response upon Sema3A binding that eventually may change the properties of the PV cell (A). Alternatively, Sema3A may act on (new) presynaptic terminals. Sema3A bound to the PNN may repel growing axons (green) away from the PV cell membrane and thereby prevent the formation of new synapses between PV-interneurons and ingrowing axons (B). Sema3A in the PNN may also “stabilize” synaptic contacts on the PV-interneuron surface by preventing local rearrangements of existing synaptic terminals (C).

Three conceivable theories which are not mutually exclusive and based on the currently available literature are outlined in Figure 6. First of all, Sema3A captured in PNN can have an effect on the PV-neuron itself by interacting with Sema3A receptors on the PV cell plasma membrane (Figure 6, model A). Secondly, as PNNs form during postnatal development, Sema3A gradually accumulates in PNN and this may repel Sema3A-sensitive fibers from the cell bodies of inhibitory interneurons thus preventing them from forming synapses on the PV-neurons (Figure 6, model B). Third, during adulthood, Sema3A in PNN could restrict the plasticity of (a subtype of) existing synapses on the PV-neuron (Figure 6, model C).

Currently, the origin of Sema3A in the PNN is not known. Studies with (genetically) labelled Sema3A in adult animals may be a first step to identify the Sema3A producing cells. Information of the cellular source is required to design future experiments to interfere with Sema3A expression and study the mechanism(s) by which Sema3A may be involved in PNN regulated plasticity.

More knowledge on the expression of Sema receptor components and their localization in PNN-positive cells and their synaptic partners is needed. The observation that net-bearing cells themselves express some components (PlexA1 and A4) of the Sema3A receptor complex is a first indication that the interneuron itself may be (one of) the target(s) of Sema3A (Figure 6, model A). PV-cells in the cat visual cortex selectively express a downstream effector of Sema3A signalling, collapsin responsive mediator protein-4 (CRMP-4) [98]. Interestingly, reorganization of the cortical projection zone following a binocular retinal lesion is accompanied by an increase in CRMP4 expression [99].

Well in line with its role during development of the nervous system, Sema3A in the PNN could repel or induce pruning of inappropriate fibers that try to establish contacts on PV-interneurons during late postnatal development (Figure 6, model B). Cortical and thalamic axons have abundant synapses on neocortical PV-interneurons but they also are sensitive to the repulsive activity of Sema3A* in vitro* [100–102]. One could hypothesize that, during development, the accumulating levels of Sema3A in the PNN reach a threshold level which can act as a selective force that only allows the strongest and/or nonreceptor carrying axons to establish or maintain synaptic contacts on the PV-interneuron and that all others are repelled or pruned. Although effects of Sema3A on the cytoskeleton restrict growth cone motility, it is currently unknown if Semaphorins in the PNN also can contribute to the stabilization of existing synapses on the PV-interneuron (Figure 6, model C). However, there is clear evidence that several members of the Semaphorin family, including Sema3A, have a central role in the formation, pruning,y and function of different types of synapses (reviewed in [10]).

NP1 is an obligatory component of the functional Sema3A receptor complex. NP1 binds to Sema3A and to Plexin and is essential for the stabilization of the Sema3A-Plexin interaction [103]. To date, we have not been able to detect NP1 in inhibitory interneurons or in synapses on the cell bodies of these neurons. This questions the validity of the idea that PNN-bound Sema3A has the capacity to act as a repulsive signal through the classical NP/Plexin receptor complex. The specific interaction of Sema3A with CS-E in PNN does raise the intriguing possibility that CS-E may stabilize the interaction of Sema3A with PlexinA which may result in functional Sema3A-PlexinA signalling independent of its interaction with NP1.

An increasing number of studies provides evidence that developmental axon guidance cues also play a significant role in synaptic remodeling and function in the adult nervous system [104]. Dysregulation of these proteins during adulthood may contribute to undesirable changes in synaptic connectivity and lead to neurological dysfunction [11]. If Semaphorins play an important role in PNN regulated neuronal plasticity, then unwanted changes in their expression could have serious consequences for the function of the nervous system. In two experimental models for epilepsy (temporal lobe and status epilepticus), dysregulation of Semaphorins is thought to contribute to the aberrant sprouting observed in the hippocampal system in these models [85, 105]. Taken together, we can also envision that controlled neutralization of Sema3A in PNN may be an important approach to enhance neuronal plasticity and functional repair after injury.
